# Diagnosis of Invasive Meningioma Based on Brain-Tumor Interface Radiomics Features on Brain MR Images: A Multicenter Study

**DOI:** 10.3389/fonc.2021.708040

**Published:** 2021-08-20

**Authors:** Dongdong Xiao, Zhen Zhao, Jun Liu, Xuan Wang, Peng Fu, Jehane Michael Le Grange, Jihua Wang, Xuebing Guo, Hongyang Zhao, Jiawei Shi, Pengfei Yan, Xiaobing Jiang

**Affiliations:** ^1^Department of Neurosurgery, Union Hospital, Tongji Medical College, Huazhong University of Science and Technology, Wuhan, China; ^2^Department of Neurosurgery, Taihe Hospital, Hubei University of Medicine, Shiyan, China; ^3^St Mary’s Hospital, Isle of Wight NHS Trust, Newport, United Kingdom; ^4^Department of Radiology, Union Hospital, Tongji Medical College, Huazhong University of Science and Technology, Wuhan, China; ^5^Department of Pathology, Union Hospital, Tongji Medical College, Huazhong University of Science and Technology, Wuhan, China; ^6^Department of Ultrasound, Union Hospital, Tongji Medical College, Huazhong University of Science and Technology, Wuhan, China; ^7^Clinical Research Center for Medical Imaging in Hubei Province, Wuhan, China; ^8^Hubei Province Key Laboratory of Molecular Imaging, Wuhan, China

**Keywords:** meningioma, brain invasion, radiomics, magnetic resonance images, peritumoral regions, prediction

## Abstract

**Background:**

Meningioma invasion can be preoperatively recognized by radiomics features, which significantly contributes to treatment decision-making. Here, we aimed to evaluate the comparative performance of radiomics signatures derived from varying regions of interests (ROIs) in predicting BI and ascertaining the optimal width of the peritumoral regions needed for accurate analysis.

**Methods:**

Five hundred and five patients from Wuhan Union Hospital (internal cohort) and 214 cases from Taihe Hospital (external validation cohort) pathologically diagnosed as meningioma were included in our study. Feature selection was performed from 1,015 radiomics features respectively obtained from nine different ROIs (brain-tumor interface (BTI)2–5mm; whole tumor; the amalgamation of the two regions) on contrast-enhanced T1-weighted imaging using least-absolute shrinkage and selection operator and random forest. Principal component analysis with varimax rotation was employed for feature reduction. Receiver operator curve was utilized for assessing discrimination of the classifier. Furthermore, clinical index was used to detect the predictive power.

**Results:**

Model obtained from BTI4mm ROI has the maximum AUC in the training set (0.891 (0.85, 0.932)), internal validation set (0.851 (0.743, 0.96)), and external validation set (0.881 (0.833, 0.928)) and displayed statistically significant results between nine radiomics models. The most predictive radiomics features are almost entirely generated from GLCM and GLDM statistics. The addition of PEV to radiomics features (BTI4mm) enhanced model discrimination of invasive meningiomas.

**Conclusions:**

The combined model (radiomics classifier with BTI4mm ROI + PEV) had greater diagnostic performance than other models and its clinical application may positively contribute to the management of meningioma patients.

## Introduction

Brain invasion (BI), described as abnormal tumor projections into the basal parenchyma, which lacks an overlapping layer of the leptomeninges, has previously been considered to possess therapeutic predictive and prognostic benefits in the management of meningiomas (1–7). The designation of central nervous system (CNS) tumors by the World Health Organization (WHO) was revised in 2016, and this entity was incorporated as a separate stand-alone classification standard for atypical meningiomas (WHO grade II) (5). As a result, the presence of BI will now significantly affect diagnosis, adjuvant care, and, finally, prognosis in these patients. Though preoperative BI detection is critically important, it continues to present challenges in clinical practice. In this respect, quantitative image analysis, or radiomics, has displayed potential in BI detection. Radiomics can automatically obtain thousands of tumor parameters from medical images centered on intensity texture and geometric features, which could then be used to demonstrate the relationship between the underlying biological mechanisms and clinical implications (8). The standard method employed in radiomics is to evaluate the internal components of tumors. Despite the fact that this technique has been considered helpful in several neoplastic diseases, it has certain limitations when dealing with BI. The main flaw is that it is unable to capture information on peritumoral regions, which is a critical element for detecting invasive growth. Malignant CNS tumors, such as glioblastoma multiforme, develop infiltrative growth patterns and invade surrounding tissues; actual tumor margins will stretch several millimeters past the radiographically detectable margins (9). The situation is comparable in invasive meningiomas, wherein diffuse (single tumor cells extending into the encircling parenchyma) and cluster-like (clustered islands or nests of tumor cells) invasive patterns are sometimes too cryptic to be recognized during medical imaging (10). When using this analytical technique, it remains important to include the peritumoral regions in the radiomics analysis to effectively identify meningioma invasiveness (11). In addition to infiltrative tumor cells, the peritumoral regions may accommodate other elements, such as normal tissue or edema, and integrating these elements for examination may limit the diagnostic capability of the radiomics (9). Hypothetically, there may be an optimal width for analyzing the peritumoral regions that ideally represent the level of tumor penetration and development patterns, while minimizing the influence of intervening factors, such as normal parenchyma. Furthermore, it would be interesting to note whether the analytical information obtained from peritumoral regions alone could perhaps be utilized for radiomics analysis, or whether the cumulative information from the intra- and peritumoral regions is more beneficial. Currently, we are not aware of any research that has attempted to address these concerns.

This study intends to evaluate the comparative performance of radiomics signatures derived from varying regions related to intratumoral region (whole tumor), peritumoral region (brain-tumor interface), and the amalgamation of the two regions (combine region) in predicting BI and to ascertaining the optimal width of the peritumoral regions needed for accurate analysis. We also used clinical, volumetric, and blood indices in the assessment, to determine if we could enhance the diagnostic strength of the envisaged models.

## Methods

### Patients

The ethics committees of the Wuhan Union Hospitals and Taihe Hospitals approved this research, and the requirement for informed consent was signed. All individual medical data were anonymized prior to analysis. We reviewed all cases of neuropathologically diagnosed meningioma, from January 2012 to April 2020, in the neurosurgical database of Wuhan Union Hospital. Patients were included in the study if the following criteria were met: (a) recently diagnosed intracranial meningiomas awaiting resection; (b) undergone relevant MRI and blood testing in the same hospital, 4 and 2 weeks prior to surgery; (c) MR images were accessible in DICOM format, enveloping T1WI and T2 fluid-attenuated inversion recovery (FLAIR) and contrast-enhanced T1-weighted (CE-T1WI) sequences; (d) MR images were free of visible artefacts or spatial distortions; (e) full blood test results were accessible in the electronic medical database system. Patients with recurrent intracranial lesions, neurofibromatosis type II, or a history of brain surgery or radiotherapy were excluded. Based on these criteria, 505 successive patients from Wuhan Union Hospital (XHH cohort) were included in the analysis, with 105 having BI and 400 without BI (NBI). Subsequently, a temporal split-sample method was used to partition the dataset into a training set of 404 patients (84% without BI) and an internal validation set of 101 patients (21% with BI).

**Figure 1:**
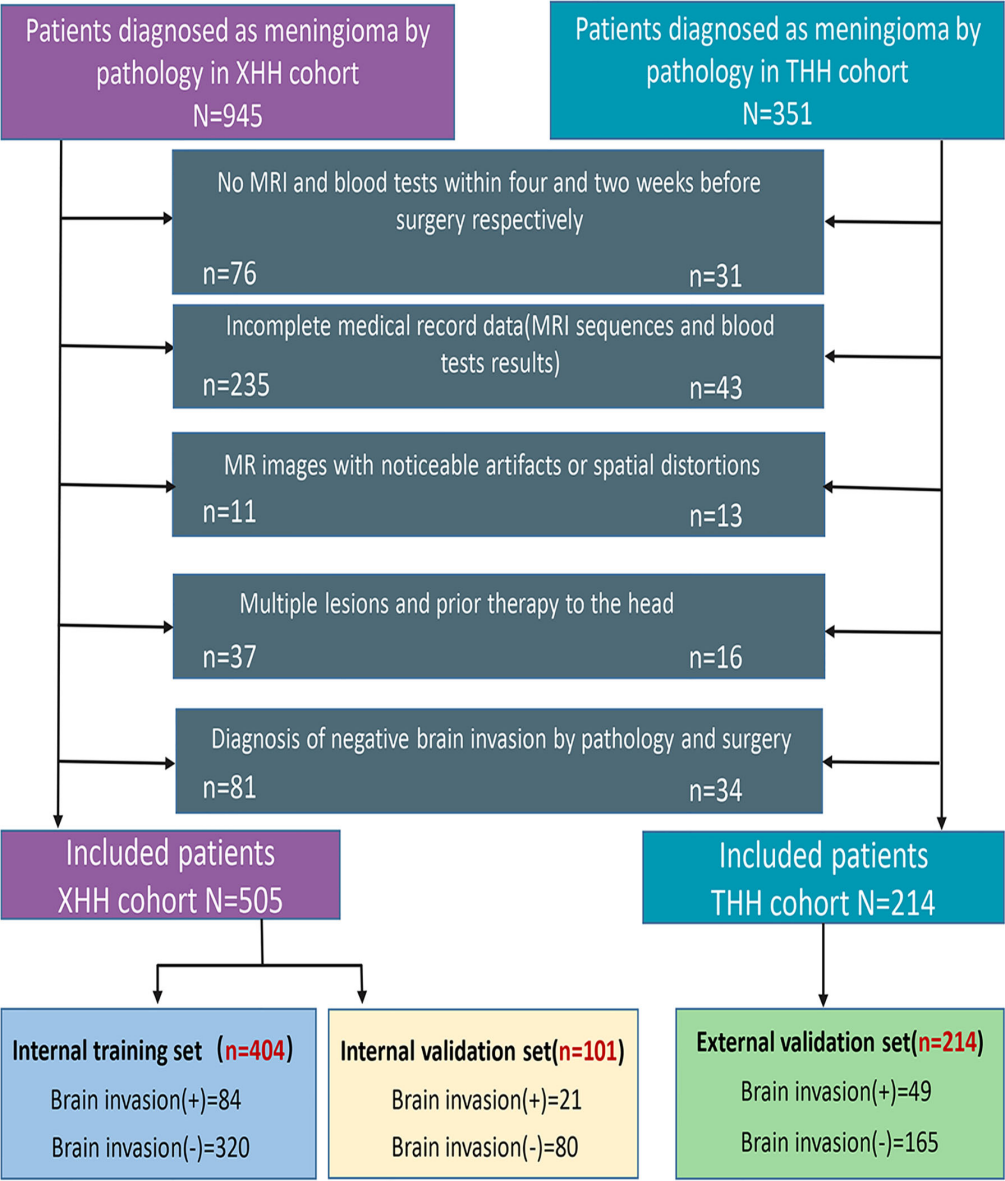
Patient selection criteria.

External validation was performed using data from Taihe Hospital (THH cohort) with a similar search technique employed. Following the implementation of the inclusion and exclusion criteria, a total of 214 successive patients were admitted, with 49 having BI and 165 being NBI. **Figure 1** displays a flow diagram describing the selection process.

Importantly, BI was evaluated retroactively and determined retrospectively, based on pathological and operative results. Meningioma specimens with adjacent CNS tissues were routinely examined for signs of invasive tumor growth by a junior neuropathologist in our centers, using the guidelines employed by Perry et al.; a senior neuropathologist later reviewed the findings to guarantee accuracy (1). We began by reviewing each patient’s pathological analysis. If there was a clear indication of BI in the study, the patient was classified as BI (105 cases); if there was brain parenchyma in the tumor specimen, but no indication of BI was found, the patient was classified as NBI (279 cases); and if there was no brain parenchyma in the specimen, the accompanying operative report was examined. When considering the operative report, if valid comments on invasive growth or breach of the pial-arachnoid border were present, the patient would be excluded from the analysis (81 cases), otherwise, the patient would be classified as NBI (121 cases). This method of analysis yielded a total of 105 cases with BI and 400 cases with NBI in the XHH cohort.

### Image Analysis

MR images in XHH and THH cohort were carried out with the aid of the 1.5T (Siemens Avanto, Erlangen, Germany) or 3.0T (Siemens Trio, Erlangen, Germany) magnetic resonance clinical scanners with a standard head coil. Images were obtained with a line of sight of 230 × 230 mm, a matrix of 512 × 512, a slice thickness of 5 mm, and a flip angle of 90°. The repetition time (TR)/echo time (TE) for T1WI and FLAIR sequences were 500/8.4 and 9,000/105 ms, respectively. MR images in dataset III was carried out with the aid of the 3.0T (Discovery MR750w, Milwaukee, WI, USA) magnetic resonance clinical scanner. Images were obtained with a line of sight of 240 × 240 mm, a matrix of 416 × 416, a slice thickness of 5 mm, and a flip angle of 90°. The TR/TE for T1WI and FLAIR sequences were 2,009/22.7 and 9,000/130 ms, respectively. CE-T1WI sequences in the two centers were obtained following the intravenous administration of 0.2 ml/kg gadopentetate dimeglumine.

FLAIR and CE-T1WI sequences were utilized for the image analysis. The publicly available software, ITK-SNAP (version 3.8.0, http://www.itksnap.org), was selected for the segmentation tasks (12). The tumor was manually segmented on CE-T1WI by one researcher (DDX, with 6 years of clinical experience in neuroimaging). Another researcher (ZZ, with 3 years of clinical expertise in neuroimaging) manually outlined peritumoral edema on FLAIR in a similar fashion; since this region also included the tumor, the volume of the peritumoral edema (PEV) was measured by deducting the magnitude of the tumor from the total volume of this outlined region. PEV divided by tumor volume gives the peritumoral edema index (PEI). A senior researcher (PFY, with 10 years of clinical expertise in neuroimaging) examined all segmentations, and when opinions differed, common ground was established through a dialog between the clinicians. During the segmentation and examination processes, researchers were blinded to the characteristic features of the subjects. Following the initial segmentation, 50 patients were randomly selected from the data pool; the latter two researchers (DDX and ZZ) resegmented the tumor on CE-T1WI to determine and evaluate inter- and intrarater reliability.

**Figure 2:**
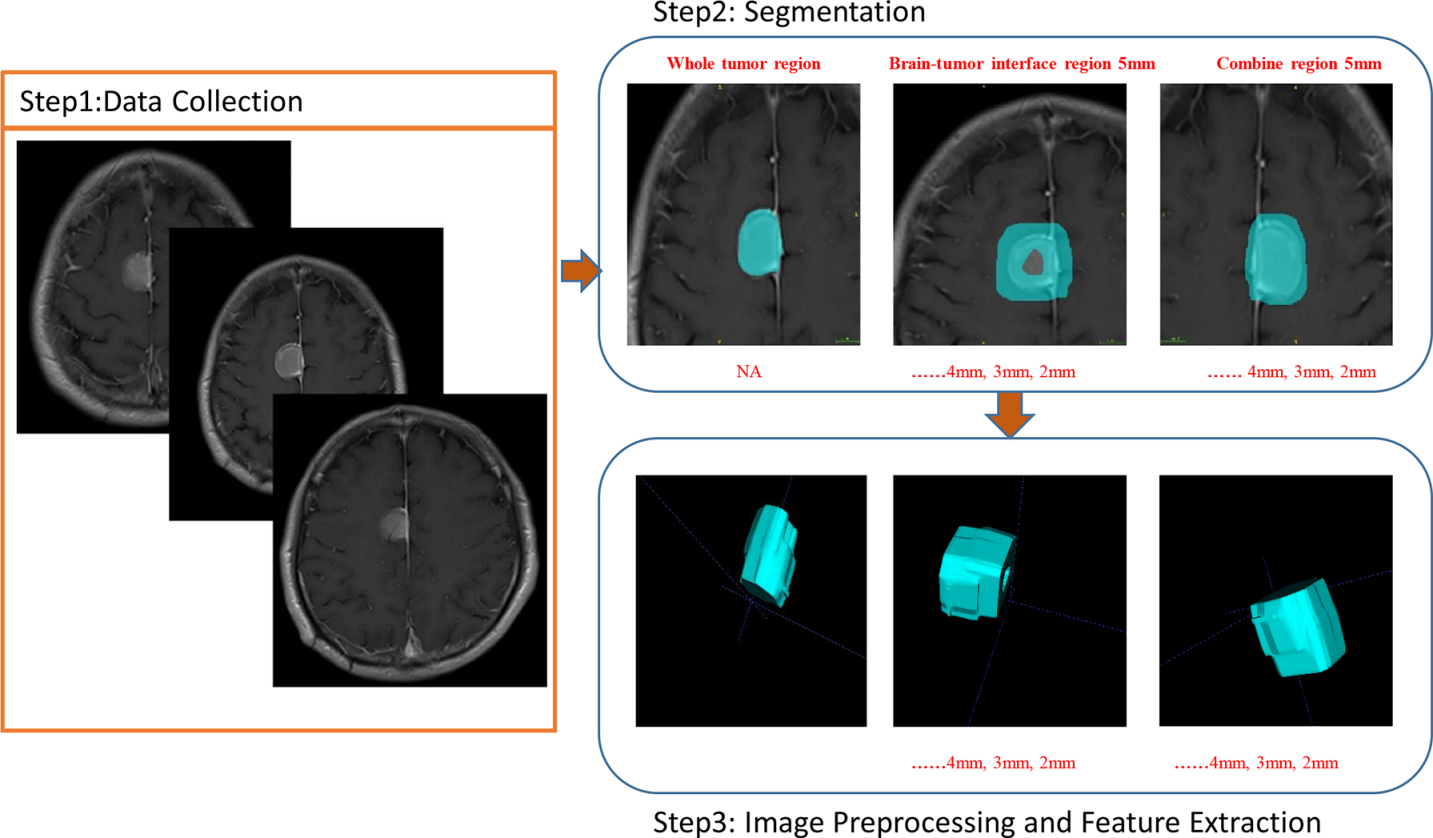
Images show the BTI, WT, and Com radiomics of invasive meningioma at CE-T1W MR image.

Relying on the tumor outline, boundary areas of the tumor were instantaneously formulated using an in-house script written in Python (version 3.8.0, https://www.python.org/). The script would concurrently move the outline N mm inward and N mm outward and generates a boundary region with a width of 2 × N mm. Each patient had four boundary areas with widths of 10, 8, 6, and 4 mm accordingly. Since meningiomas are commonly present around the skull, we streamlined these areas by manually discarding the regions adjacent to the skull to reduce the possible effect of this component on eventual image processing (investigator DDX). Investigator PFY visibly inspected all streamlined segmentations. Furthermore, we merged segmentations of the boundary areas with tumor segmentation using the same script, resulting in three new equivalent segmentations for each subject. These new segmentations consisted of information on the internal components and the surrounding regions of the tumor and thus could constitute an alternate method for detecting BI (**Figure 2**).

Radiomics features were retrieved through pyradiomics, an open-source python package (version 3.0.0, https://github.com/AIM-Harvard/pyradiomics) (13). The volume of the ROI is calculated from the triangle mesh of the ROI, which could be obtained with a ready-to-use function provided in pyradiomics. Images were preprocessed leading up to the retrieval, which focused on normalization, discretization, and resampling to a 3 × 3 × 3 isotropic voxel size. These measures were intended to enhance the efficiency and reliability of radiomics analysis and were recommended as an aspect of the methodology by the developers of the package (14, 15). Three classes of characteristics were calculated. First-order statistics (*N* = 18) define the histogram of voxel intensity values embedded inside the region of interest (ROI) *via* fundamental and popularly utilized metrics. Geometric features (*N* = 14) described the 3D form and scale of the ROI and were measured only on the 3D mask of the ROI (i.e., independent from the gray-level intensity distribution in the ROI). Textural features characterizing patterns or spatial distribution of voxel intensities were determined from the gray-level co-occurrence matrix (GLCM, *N* = 21), gray-level size zone matrix (GLSZM, *N* = 16), gray-level run length matrix (GLRLM, *N* = 16), neighboring gray tone difference matrix (NGTDM, *N* = 5), and gray-level dependence matrix (GLDM, *N* = 14). In addition to the original image, 10 derived images were produced by adding LoG or Wavelet filters. Notwithstanding, a total of 1,015 features were retrieved for each patient.

### Blood Indices

Blood samples were obtained within the 2 weeks prior to the surgery. Where several samples were present, the one taken nearest to the time of surgery was used. Absolute counts of leukocytes, erythrocytes, hemoglobin, platelets, neutrophils, lymphocytes, monocytes, albumin, and fibrinogen were evaluated. Consequently, we evaluated the neutrophil-to-lymphocyte ratio (NLR), derived NLR, neutrophil/[leukocyte-neutrophil] (dNLR), platelet-to-lymphocyte ratio (PLR), systemic immune-inflammation index (SII), monocyte-to-lymphocyte ratio (MLR), and prognostic nutritional index (PNI). The detailed descriptions and explanations of these indices have been illustrated earlier (16–22).

### Feature Selection and Modeling

Firstly, the raw values of all radiomics-extracted features were scaled from 0 to 1 as

$$X_{i}^{΄}=\frac{X_{i}-X_{\min}}{X_{\max}-X_{\min}}$$

where $X_{i}^{΄}$ indicates the scaled *i*th value of variable *X*. *X*_min_ and *X*_max_ indicate the minimum and maximum value for variable *X*, respectively.

**Figure 3:**
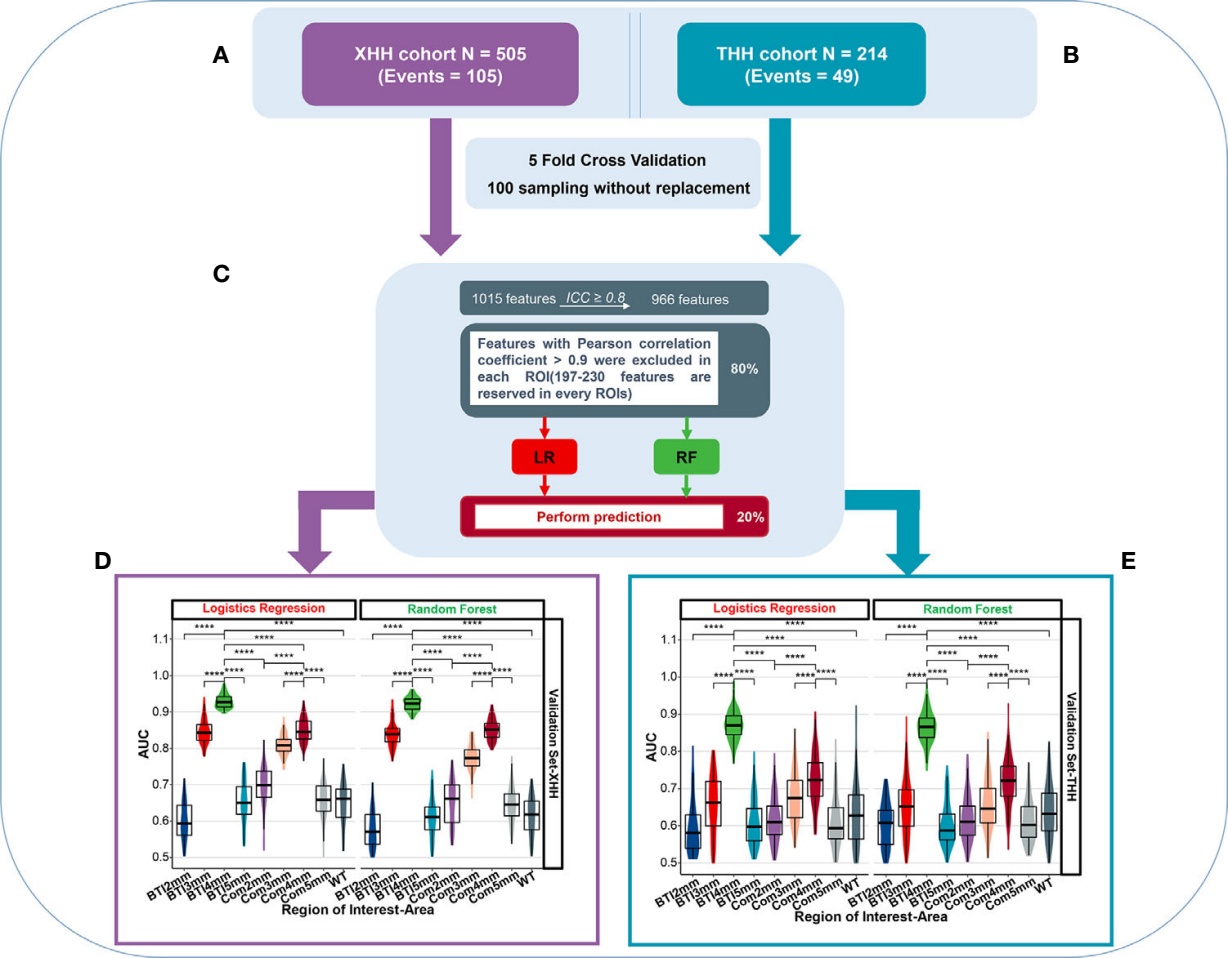
The overall predictive performance of invasive meningioma in different ROI areas between two independent cohort. **(A)** Patients were split into the XHH cohort (*n* = 505) and the THH cohort (*n* = 214). **(B)** During feature selection and classifier establishment, fivefold cross-validations of 100 sampling without replacement were used. **(C)** Schematic representation of feature selection and classifier building. **(D, E)** Violin plot of the AUC value distribution of different ROI areas in the XHH cohort (left side) and the THH cohort (right side). ****p < 0.0001.

The intraclass coefficient (ICC) was applied to assess the reproducibility of imaging metrics measured by different radiologists. As **Figure 3C** shows, only features with ICC ≥0.8 were maintained for further feature selection processing. We then applied least absolute shrinkage and selection operator (LASSO) (23) by a 10 cross-fold cross-validation, to further filter the variables, followed by ranking the importance of these variables and selecting the corresponding variables according to the events per variable principle to construct logistic regression models and random forest models, respectively. In addition, principal component analysis (PCA) (*R package version 2.1.3*, https://CRAN.R-project.org/package=psych) (24) with varimax rotation was employed for feature reduction, in an effort to retain more variance and reduce redundancy of the variables.

### Statistic Analysis

Quantitative variables are reported as mean ± SD and median (IQR), while describing variables as counts and percentages. Comparisons of frequency distributions between subgroups of categorical variables were made using the *χ*^2^ test or the exact Fisher’s test, while the *t*-test or Wilcoxon’s test was used for comparisons between numerical variables. Spearman’s correlation test was used to evaluate the collinear correlations between features.

Receiver operator curve (ROC) was utilized for assessing discrimination of the classifier; area under curve values (AUC) were calculated (25). Multiple hypothesis testing using Benjamini and Hochberg’s (BH) method was used to control false-discovery rate (FDR) for comparison between groups. The performance of predictors was expressed by its accuracy, sensitivity (i.e., true positive rate), and specificity (i.e., true negative rate), positive predictive value (PPV), and negative predictive value (NPV). Precision-recall curve (PRC) was drawn to further evaluate the model performance under the condition of sample proportion imbalance (**Figure S3**). Decision curve analysis (DCA) is a method for assessing the benefits of diagnostic tests that covers a range of patient preferences for receiving under- and overtreatment risk in order to make decisions about test selection and use (26).

Statistical analyses were performed using R software (version 4.0.1, https://www.r-project.org). Statistical significance was fixed at *p* < 0.05 (*α* = 0.05).

## Results

### Patient Characteristics

**Table 1** displays patient characteristics and contextual information. In both the XHH and THH cohorts, there was no substantial variation in age or sex among the invasive and noninvasive groups (*p* = 0.15; 0.84). High-grade meningiomas (grades II and III) were much more prevalent in the invasive group in both cohorts. There were no statistically significant variations in the laboratory research index among the invasive and noninvasive groups. The distribution of tumor and peritumoral edema volume was distorted across cohorts, with invasive meningiomas having a greater median volume in tumor and peritumoral edema (*p* < 0.001).

**Table 1:** Baseline characteristics of the patients in the different cohorts.

	XHH (training and internal validation cohorts)			THH (external validation cohort)		
	Noninvasive	Invasive	*p*	Noninvasive	Invasive	*p*
*N*	400	105		165	49	
Age						
Median [IQR]	53.0 [47.0, 60.0]	55.0 [49.0, 61.0]	0.109	52.0 [47.0, 60.0]	54.0 [48.0, 60.0]	0.146
Sex (%)						
Male	81 (20.2)	38 (36.2)	0.001	45 (27.3)	12 (24.5)	0.839
Female	319 (79.8)	67 (63.8)		120 (72.7)	37 (75.5)	
WHO grade (%)						
I	372 (93.0)	65 (61.9)	<0.001	156 (94.5)	31 (63.3)	<0.001
II	28 (7.0)	33 (31.4)		7 (4.2)	17 (34.7)	
III	0 (0.0)	7 (6.7)		2 (1.2)	1 (2.0)	
Laboratory test, (median [IQR])						
WBC (×10^9^/L)	6.18 [4.90, 8.84]	6.22 [4.95, 7.82]	0.646	5.77 [4.70, 7.32]	5.55 [4.33, 8.13]	0.587
Erythrocyte (×10^9^/L)	4.22 [3.83, 4.56]	4.25 [3.93, 4.53]	0.773	4.22 (0.52)	4.14 (0.52)	0.380
Hemoglobin (g/L)	125 [115, 135]	126 [117, 137]	0.446	126 [116, 137]	127 [115, 135]	0.708
Platelet (×10^9^/L)	195 [155, 238]	202 [162, 235]	0.667	197 [159, 238]	189 [161, 236]	0.660
Neutrophil (×10^9^/L)	3.74 [2.70, 6.79]	3.62 [2.78, 5.58]	0.714	3.50 [2.58, 5.31]	3.33 [2.49, 5.61]	0.750
Lymphocyte (×10^9^/L)	1.56 [1.04, 1.97]	1.52 [1.08, 1.95]	0.820	1.64 [1.26, 1.95]	1.52 [1.10, 1.80]	0.342
Monocyte (×10^9^/L)	0.38 [0.28, 0.49]	0.42 [0.33, 0.56]	0.003	0.35 [0.28, 0.47]	0.37 [0.32, 0.44]	0.416
Albumin (g/L)	40.1 [36.0, 43.0]	38.8 [35.6, 42.1]	0.101	40.6 [37.9, 42.8]	40.1 [38.3, 43.3]	0.684
FIB (g/L)	2.89 [2.52, 3.33]	2.89 [2.62, 3.33]	0.629	3.10 [2.75, 3.65]	3.11 [2.79, 3.69]	0.943
Magnetic resonance imaging						
TV (median [IQR]; ml)	21.8 [7.7, 46.1]	30.1 [13.9, 54.4]	0.021	16.6 [7.9, 37.3]	36.7 [16.7, 62.9]	<0.001
PEV (median [IQR]; ml)	16.0 [4.6, 51.3]	51.3 [17.4, 114.3]	<0.001	8.8 [2.9, 29.3]	41.9 [12.0, 83.1]	<0.001

IQR, interquartile range; WBC, white blood cell; FIB, fibrinogen; TV, tumor volume; PEV, peritumoral edema volume.

### Comparison of Performance Across ROI Areas

#### BTI4mm-Based Radiomics Features Showed the Best Diagnostic Performance Independently in Both the XHH and THH Cohorts

Patients has been devided into XHH cohort (**Figure 3A**) and THH cohort (**Figure 3B**). Prior to the radiomics analysis, we exempted 49 radiomics features with an intraclass correlation coefficient <0.8 (**Figure S2**), representing a broader margin of variation under modest segmentation disparities. The residual 966 features were vetted by Spearman’s correlation test and features with Spearman’s correlation coefficient >0.9 were excluded in the XHH and THH cohorts, respectively (**Figure 3C**). The features included are summarized in **Table S1**. Considering the number of events per variable (EPV), the nine top features in the XHH cohort and three features for the THH cohort were chosen in every iteration. **Figures 3D, E** depicts the distribution of AUCs for nine ROIs after 100 iterations. The average AUC of the BTI4mm model in the XHH cohort (**Figure 3D**) is much greater than that of the other models based on various ROIs, both by logistic regression (AUCmean = 0.929) and random forest algorithms (AUCmean = 0.922). The THH cohort (**Figure 3E**) also exhibits the same pattern. Furthermore, the XHH cohort BTI4mm model predicted disease invasiveness more effectively than the THH cohort (AUCmean is 0.870 and 0.865 for LR and RF, respectively).

#### Feature Reselection and Further Comparison of Radiomics Model

**Figure 4:**
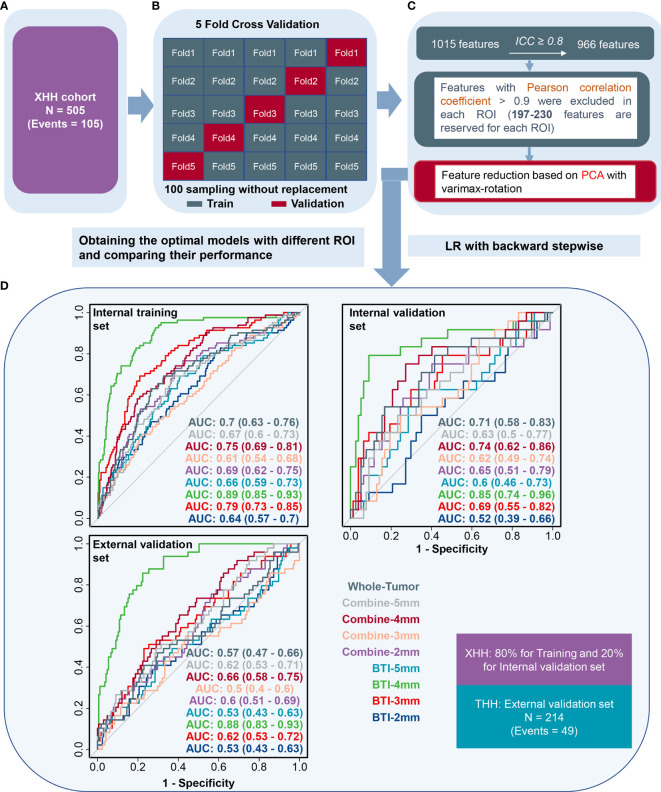
The receiver operating characteristic curves when applying the backward stepwise LR algorithm. **(A)** Patients were split into the XHH cohort (*n* = 505). **(B**, **C)** Feature selection, reduction and classifier establishment. **(D)** After feature reselection and PCA dimensional reduction, models were obtained based on principal components.

To validate the robustness of the initial results, we split the XHH cohort into the training and internal validation sets and used the THH cohort as the external validation set (**Figure 4**). After Spearman’s correlation test for both cohort, we preprocessed the radiomics features of each ROI among the nine ROIs from BTI2mm to the entirety of the tumors, we maintained 230, 225, 216, 206, 197, 199, 197, 209, and 218 features in that sequence, with the comprehensive features demonstrated in the **Table S3**. Eventually, PCA with varimax rotation was implemented to feature mitigation and eight principal components such as PC1, PC5, PC2, PC3, PC6, PC7, PC11, and PC10were acquired in the BTI4mm model. Other PCA characteristics of the ROI areas are represented in the **Figure S4**. As previously stated, the AUC values of the models constructed by LR and RF are identical (**Figures 3D, E**). Given the simplicity of assessing the model, we picked the backward stepwise LR algorithm to design models for these eight PCA indicators.

As illustrated in **Figure 4D**, BTI4mm has the maximum ROC-AUC in the training set (0.891 (0.85, 0.932)), internal validation set (0.851 (0.743, 0.96)), and external validation set (0.881 (0.833, 0.928)) and displayed statistically significant results. The PRC-AUC in the training set is 0.709, internal validation set is 0.736, and external validation set is 0.675 (**Figure S3**). P-Value of AUCs in violin plot (**Figures 3D, E**), peritumoral edema volume, and peritumoral edema index and the nine models from different regions (**Figure 4D**) were adjusted, and the adjusted *p*-value was statistically significant. Corresponding adjusted *p*-values between nine models are shown in **Table S2**.

After extensive analysis, BTI4mm was found to be the best-predictive radiomics model to put it briefly.

### Clinical Predictors for Disease Invasion

As displayed in **Table 1** and **Figure S5**, there were significant variations in peritumoral edema volume and peritumoral edema index. In the external validation sample, PEV performed better as an independent predictor, with an AUC of 0.71, especially when compared with PEI (AUC, 0.59).

### Combined Models

**Figure 5:**
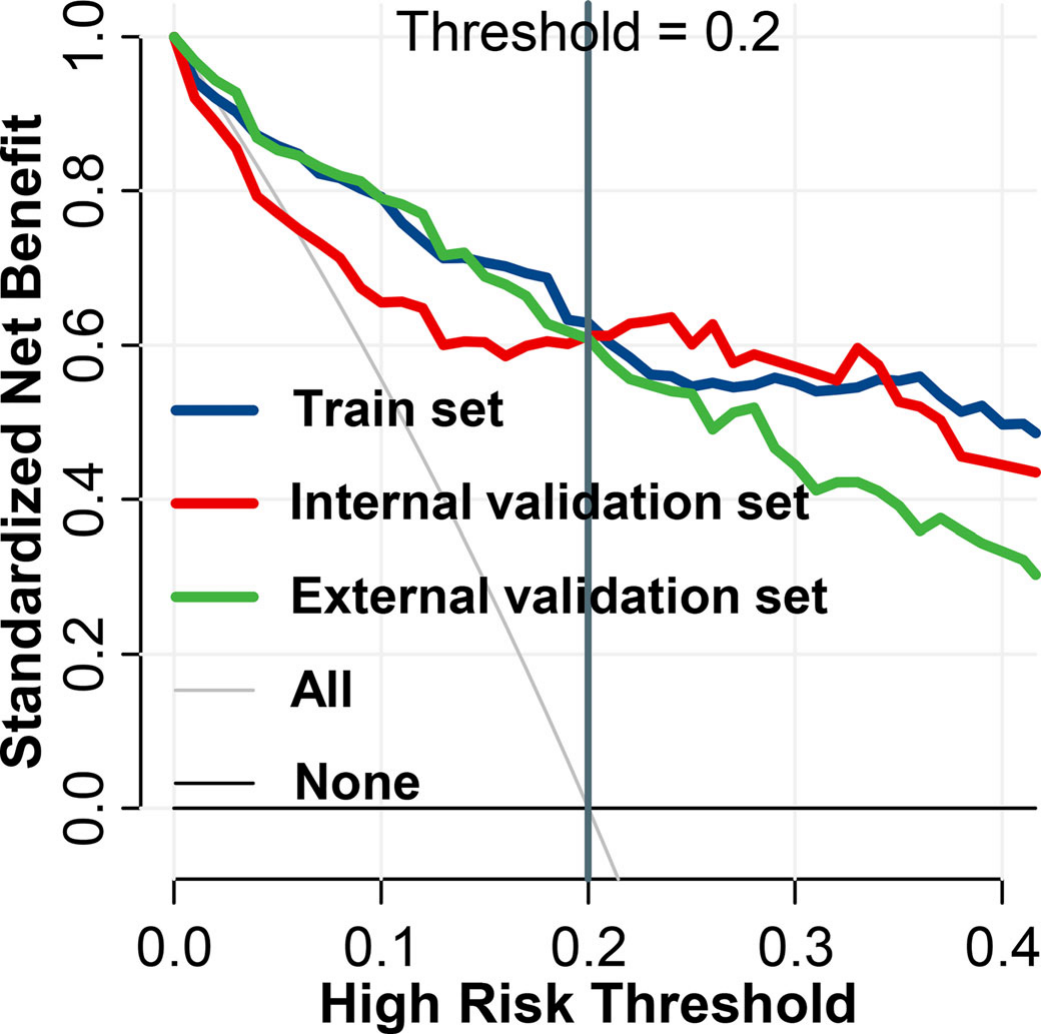
Decision curve analysis of BTI4mm model for invasion diagnosis. For the training and validation sets, the net benefit curve is shown. When threshold probability reached 0.20, the clinical benefit of the models was the greatest.

**Figure 6:**
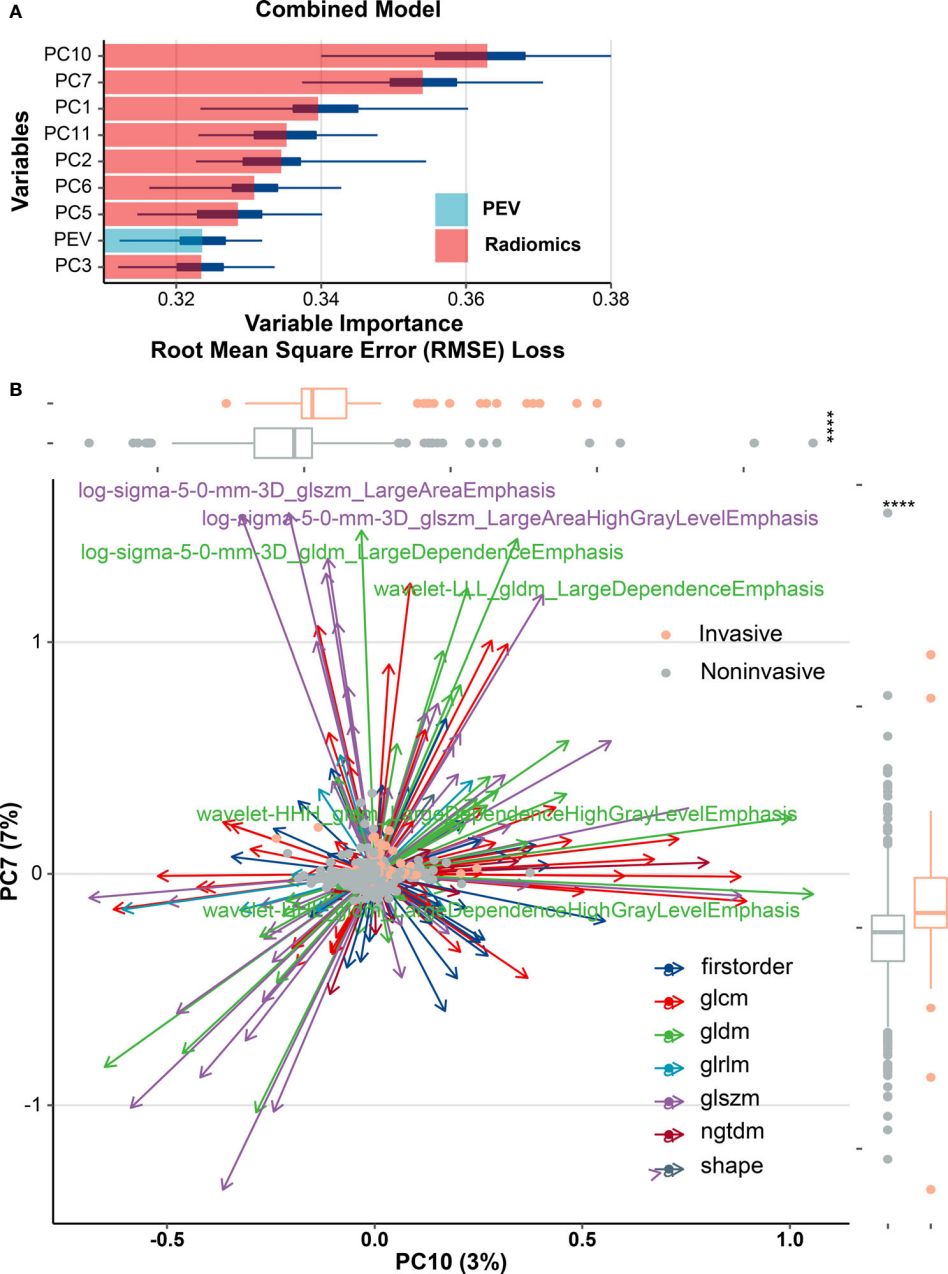
Multiple types of radiomics features associated with brain invasiveness in the combined model. **(A)** Variable importance for classification of event in combined model. **(B)** Radiomics feature-based principal component analysis (PCA) of tumor invasion. PCA shows that PC10 and PC7 are almost able to distinguish the invasive and noninvasive groups of meningiomas and the multivariate variation of different radiomics features of PC10 and PC7. A total of six features including gray-level co-occurrence matrix (GLCM) and gray-level dependence matrix (GLDM) were subjected to PCA, and those features with variable loading for PC7 ≥0.7 or PC10 ≥0.5 were shown as major contributors. Box plots show the overall distribution of PC10 and PC7 scores within tumor invasion (Wilcoxon rank-sum test). PEV, peritumoral edema volume; ****p < 0.0001.

As per the maximum clinical benefit recommended by DCA curves (**Figure 5**), the cutoff value was set as 0.20, and the comprehensive diagnostic performances are demonstrated in **Table 2**. The radiomics-based model outperformed the clinical parameter-based model in both internal and external set discrimination of invasive meningioma on ROC study. The addition of PEV to radiomics features enhanced the model discrimination of invasive meningiomas. **Figure 6A** depicts the ranking of variable priority for discriminating invasive meningioma in the combined model, and essential radiomics parameters are encapsulated in a heatmap (**Figure 6B**). Between these clusters, radiomics features that align exceptionally well with PC10 and PC7, and provide the best diagnostic results, are almost entirely generated from GLCM, GLDM, and GLSZM statistics.

**Table 2:** Diagnostic performance of classifiers.

Parameter	Combined model	Clinical model	Radiomics model
	Peritumoral edema volume + interface radiomics (4 mm)	Peritumoral edema volume	Interface radiomics (4 mm)
Training sets			
TN/FP/FN/TP	255/68/13/68	248/72/43/41	253/70/13/68
AUC	0.898 (0.857, 0.939)	0.702 (0.639, 0.765)	0.891 (0.85, 0.932)
Accuracy	0.8 (0.757, 0.837)	0.715 (0.669, 0.759)	0.795 (0.752, 0.833)
Sensitivity/recall	0.84	0.488	0.84
Specificity	0.789	0.775	0.783
PPV/precision	0.5	0.363	0.493
NPV	0.951	0.852	0.951
NRI (categorical)	Ref	NA	−0.04 (−0.10, −0.03)
NRI (continuous)	Ref	NA	−0.43 (−0.66, −0.19)**^†^**
IDI	Ref	NA	−0.03 (−0.06, 0)**^†^**
Internal validation sets			
TN/FP/FN/TP	64/13/4/20	64/16/15/6	64/13/5/19
AUC (95% CI)	0.854 (0.745, 0.963)	0.608 (0.468, 0.749)	0.851 (0.743, 0.96)
Accuracy	0.832 (0.744, 0.899)	0.693 (0.593, 0.781)	0.822 (0.733, 0.891)
Sensitivity/recall	0.833	0.286	0.792
Specificity	0.831	0.8	0.831
PPV/precision	0.606	0.273	0.594
NPV	0.941	0.81	0.928
NRI (categorical)	Ref	NA	−0.01 (−0.1, 0.07)
NRI (continuous)	Ref	NA	0.21 (−0.15, 0.57)
IDI	Ref	NA	0.02 (0, 0.06)
External validation sets			
TN/FP/FN/TP	124/41/7/42	140/25/30/19	131/34/10/39
AUC	0.885 (0.839, 0.932)	0.709 (0.628, 0.79)	0.881 (0.833, 0.928)
Accuracy	0.776 (0.714, 0.83)	0.743 (0.679, 0.8)	0.794 (0.734, 0.846)
Sensitivity/recall	0.857	0.388	0.796
Specificity	0.752	0.848	0.794
PPV/precision	0.506	0.432	0.534
NPV	0.947	0.824	0.929
NRI (categorical)	Ref	NA	0.01 (−0.08, 0.11)
NRI (continuous)	Ref	NA	0.09 (−0.37, 0.19)
IDI (95% CI)	Ref	NA	0 (−0.03, 0.02)

P < 0.05. Ref, Reference; Na, Not applicable.

## Discussion

This multicenter study analyzed the BTI2mm, BTI3mm, BTI4mm, BTI5mm, Com2mm, Com3mm, Com4mm, Com5mm, and WT ROI areas independently to formulate and confirm nine radiomics signatures to forecast brain invasion in meningioma patients. We discovered that BTI4mm ROI had a higher ROC-AUC and PRC-AUC, particularly compared with other models both in the training set, internal validation set, and external validation set and acquired the highest AUC value of 0.891, 0.851, and 0.881, respectively. Compared with other ROIs, the diagnostic performance of the BTI4mm model in the THH cohort was not as high as in the XHH cohort after 100 iterations. However, both provided better classification performance independently. Furthermore, peritumoral edema volume enhanced the diagnostic performance of the BTI4mm radiomics model as an autonomous clinical predictor.

A subsequent study stated that brain-tumor interface MRI features in the combination of multisequences (T1C and T2) with a boundary of 5 mm (as in our research) allow forecasting of brain invasion in meningioma, with a recorded AUC of 0.82 in the corresponding internal validation set (11). That being said, in the results, the writers employed only the T1C phase to train radiomics models and obtained a lower AUC value for BTI5mm (AUC, 0.72). In the research of Zhang et al., combining T1C and T2 models resulted in enhanced discrimination capacity by 4.77% and 6.34%, accordingly, as contrasted to the T1C and T2 models (27). Moreover, in the external validation set, radiomics models combining T1C and T2 yielded a higher AUC (0.796) than a single T1C sequence WT model (AUC, 0.72). T1C images are not only typically used to represent tumor boundary and blood flow but can also be used to assess the level of tumor invasiveness (28). T2 imaging is more effective at measuring edema and is typically used on water-rich tissue (29). As a result, multisequence radiomics models may be more sensitive in display detail about tumors and have a higher diagnostic capacity than T1C and T2 sequences alone. Notably, in the analysis, TBTI4mm features derived from only the T1C sequence demonstrated outstanding diagnostic efficiency with an AUC of 0.851 in the internal validation set, which was higher than the multisequence radiomics models in the previous two tests. While having an AUC of merely 0.881, BTI4mm was the best-performing model compared with the other ROIs in the external validation set.

We considered potential reasons as to why the BTI4mm ROI area demonstrated the overall best-predictive effectiveness in diagnosing invasive meningioma. Some research centered on detecting glioma microstructural heterogeneity using sophisticated MRI, which implicitly showed the infiltrating extension relative to standard white matter (30–32). Niha et al. claimed that radiomics features extracted from intranodular and perinodular regions of nodules can distinguish nonsmall-cell lung cancer adenocarcinomas from benign granulomas at noncontrast CT, and the most predictive features were found to be within an immediate distance of 5 mm from the nodule (33). In light of our observations, we concluded that the 4-mm extension boundary within and outside the tumor contour could have an increased level of heterogeneity for most invasive meningioma. Normal brain around the tumor may reveal tumor cells trying to infiltrate the surrounding parenchyma and lacking an intervening layer of leptomeninges (10), which is essentially distinct from noninvasive meningiomas. Recent studies have focused on the correlation between meningioma pathological grade and radiomics features. The research of Park et al., for example, indicated that MRI-based radiomics features enabled the differentiation of meningioma grading (34). Our previous study also reflected the potential value of preoperative MRI texture and shape analysis in grading meningiomas (35). Maria Caffo’s study indicated that brain invasion significantly influence the DFS of Simpson’s grade I meningioma. The prognosis of meningioma is determined by many factors, besides brain invasion, high mitotic index and sheeting also indicated poor outcome. Researchers deemed that atypical meningioma should be diagnosed mainly based on high mitotic index or brain invasion, which is more associated with recurrence than minor criteria (34). Until now, there have been very few studies on the invasive expansion extent of meningiomas. In this research, the average tumor volume of the XHH and THH cohorts was 21 and 23 ml, respectively. It may serve to reflect the aggregate volume of meningiomas in large-scale hospitals in China (**Figure S1**).

Nevertheless, owing to the graded diagnosis and care program of the country, meningioma size in primary medical units is smaller than in high-level hospitals. As a result, BTI4mm ROI could not accurately depict the maximum heterogeneity in primary care hospitals. We considered whether there was a proportional association between the extent of meningioma infiltration and tumor size that deserved further investigation.

PC10 and PC7 have a significant weighting coefficient in variable importance analysis and have a robust predictive strength for diagnosing invasive meningioma. The radiomics feature-based principal component analysis revealed that the highest output is almost entirely PC10, PC7, and PC1 elements, which are almost entirely generated from the GLCM, GLDM, and GLSZM statistics. The result of GLCM and GLDM is consistent with the research of Leehi et al. (11). PEV boosted the AUC of the radiomics model by about 0.003 in the external validation set and 0.007 in the training set as an independent predictor for an AUC of about 0.702. The eight components of the unified model included radiomics solid features to the fullest degree possible, assisting in avoiding heterogeneous information loss. This might be part of the reason why T1C sequences solely facilitate an optimal classifier performance.

The use of radiomics methods to implicitly portray tumor infiltration is significant for clinical transformation applications, mainly in determining the presence of an invasive meningioma. The gold standard for BI assessment remains neuropathological investigations (1, 7). Unfortunately, this has the inherent flaw of only being able to make the correct diagnosis a few days following surgery. Predicting BI before surgery is of great importance in clinical practice for many reasons. For starters, information on tumor invasiveness may assist in determining future management and considering surgical plans for patients with minor and asymptomatic lesions. Though such cases typically do not necessitate early excision, signs of invasiveness can necessitate closer monitoring or even earlier elimination. Secondly, some research suggests that BI is a significant indicator of perioperative complications (2–4). For example, if it can be shown that patients with BI are genuinely at greater risk of perioperative seizures, antiepileptic treatment could be commenced in a timely fashion. Finally, it has been proposed that surgical procedures and the degree of histological sampling may affect the neuropathological diagnosis of BI (1, 36–38). Preoperative BI awareness should be demonstrated in communication among neurosurgeons and neuropathologists, enhancing neuropathological diagnosis precision by taking strategic measures.

Since the publication by Perry et al. in 1997, BI has been constantly described as the presence of tumor cells within the adjacent brain tissue without a dividing connective tissue layer (1). As per this definition, conclusive diagnosis of BI depends entirely on histopathological evaluation, which requires the existence of adjacent brain parenchyma. Consequently, since in reality most meningioma specimens lack brain parenchyma, definitive diagnosis is generally challenging. The fundamental cause for the absence of CNS tissue is that neurosurgeons tend to keep the arachnoid membrane intact wherever possible to prevent neurological injury. Another possible explanation is nonstandardized intraoperative tumor sampling (37). Several operation-related variables, including partial resection and the use of a cavitron ultrasonic surgical aspirator (CUSA), appear to be relevant to the reduced ease of access to CNS tissue. Timme et al. demonstrated that microsurgery nuances could affect the existence of CNS parenchyma on meningioma specimens and thus change the efficiency of BI detection (38). Histopathological sampling methods may also affect the evaluation of BI (7). As a result, the existence of BI is most likely underreported.

To remedy this shortcoming, two recent studies (37, 39) sought to ascertain the advantages of a hybrid strategy that contained knowledge from both operative and pathological observations. Their early findings showed that the combined method had the ability to transcend the shortcomings of sole neuropathological evaluation and may be of considerable therapeutic benefit. In light of these findings, we embraced identical combined standards to evaluate BI in the current study. Importantly, for patients whose tumor specimens revealed no ordinary CNS tissues, the resulting operative reports were evaluated. If the operative reports described the presence of BI, it is difficult to confirm whether BI had in fact occurred, and this makes it impracticable to ascertain BI without neuropathological verification. To prevent potential bias, we agreed to eliminate these cases during the patient-selection stage. These combined criteria should be able to reduce the effect of incorrectly classifying cases on our analysis. It can also help to understand the comparatively high proportion of BI in the current study.

There are some drawbacks to this analysis that should be stated. Owing to the retrospective nature of the results, uncertain confounding variables may be present. Second, MRI images were collected using a range of parameters and devices, which may affect the efficiency and robustness of the radiomics features. To minimize this effect, we used widely suggested preprocessing pipelines prior to the actual study. Third, a manual technique was employed for tumor segmentation; the critical reason for this was that comparing segmentation approaches was not the primary objective of this work. A manual approach could, in our view, yield precise delineations of meningiomas with adequate time and labor.

Furthermore, the ICC analyses demonstrated the accuracy of the manual delineations. Fourth, we only considered radiomics features extracted from CE-T1WI sequences, while other related research used or proposed T2WI, FLAIR, and DWI sequences. This may be viewed as a limitation in this study; however, reaching satisfactory prediction accuracy with fewer MR sequences may encourage clinical implementation of the proposed model.

Lastly, we only considered the functionality of the variables stated previously, in predicting BI in meningiomas; a more instinctive and important question would be to consider whether these variables are correlated to patient prognosis, particularly given the ongoing debate about the true prognostic value of BI. We intend to gather such data in order to answer this question in future studies.

## Conclusion

In conclusion, the study found that the radiomics classifier with BTI4mm ROI had greater diagnostic performance than the other eight radiomics models: BTI2mm, BTI3mm, BTI5mm, com2mm, com3mm, com4mm, com5mm, and WT. PEV strengthened the diagnostic capacity of the radiomics classifier in detecting invasive meningioma. Further prospective multicenter research is required to determine the best BTI extension for detecting invasiveness in various tumor size groups.

## Data Availability Statement

Data supporting the conclusions of this article are available on reasonable request from any qualified investigator.

## Ethics Statement

The studies involving human participants were reviewed and approved by the study protocol was approved by the Ethics Committee of the Wuhan Union Hospital and Taihe Hospital (NO. 2021-0098-01). The patients/participants provided their written informed consent to participate in this study. Written informed consent was obtained from the individual(s) for the publication of any potentially identifiable images or data included in this article.

## Author Contributions

Conception and design: XBJ, PFY, and JWS. Collection and assembly of the data: DDX, ZZ, JL, XW, PF, JJW, XBG, and JMLG. Development of the methodology: JWS, DDX, and PFY. All authors contributed to the article and approved the submitted version.

## Funding

This study has received funding from the National Natural Science Foundation of China (81974390), and the funders had role in study design and data collection.

## Conflict of Interest

The authors declare that the research was conducted in the absence of any commercial or financial relationships that could be construed as a potential conflict of interest.

## Publisher’s Note

All claims expressed in this article are solely those of the authors and do not necessarily represent those of their affiliated organizations, or those of the publisher, the editors and the reviewers. Any product that may be evaluated in this article, or claim that may be made by its manufacturer, is not guaranteed or endorsed by the publisher.

## Supplementary Material

Supplementary Figure 1 Violin plot of the PEV and TV value distribution of different ROI areas in the XHH cohort and THH cohort. PEV, peritumoral edema volume; TV, tumor volume.

Supplementary Figure 2 Heatmap of radiomics features with an intraclass correlation coefficient <0.8.

Supplementary Figure 3 The predictive performance of models under the precision-recall curve. Internal training set **(A)**; internal validation set **(B)**; external validation set **(C)**. *****p* < 0.0001.

Supplementary Figure 4 Scree plot with parallel analysis for identifying the number of principal components to be kept. The blue cross indicates a principal component, and those above the red dashed line are candidate components.

Supplementary Figure 5 The receiver operating characteristic curves of PEV and PEI derived from training and validation sets. *****p* < 0.0001.
