# Supplementary figures and images for: Diagnosis of Invasive Meningioma Based on Brain-Tumor Interface Radiomics Features on Brain MR Images: A Multicenter Study
Source: Front Oncol. 2021 Aug 20;11:708040. doi: 10.3389/fonc.2021.708040 (PMC8422846; doi:10.3389/fonc.2021.708040)

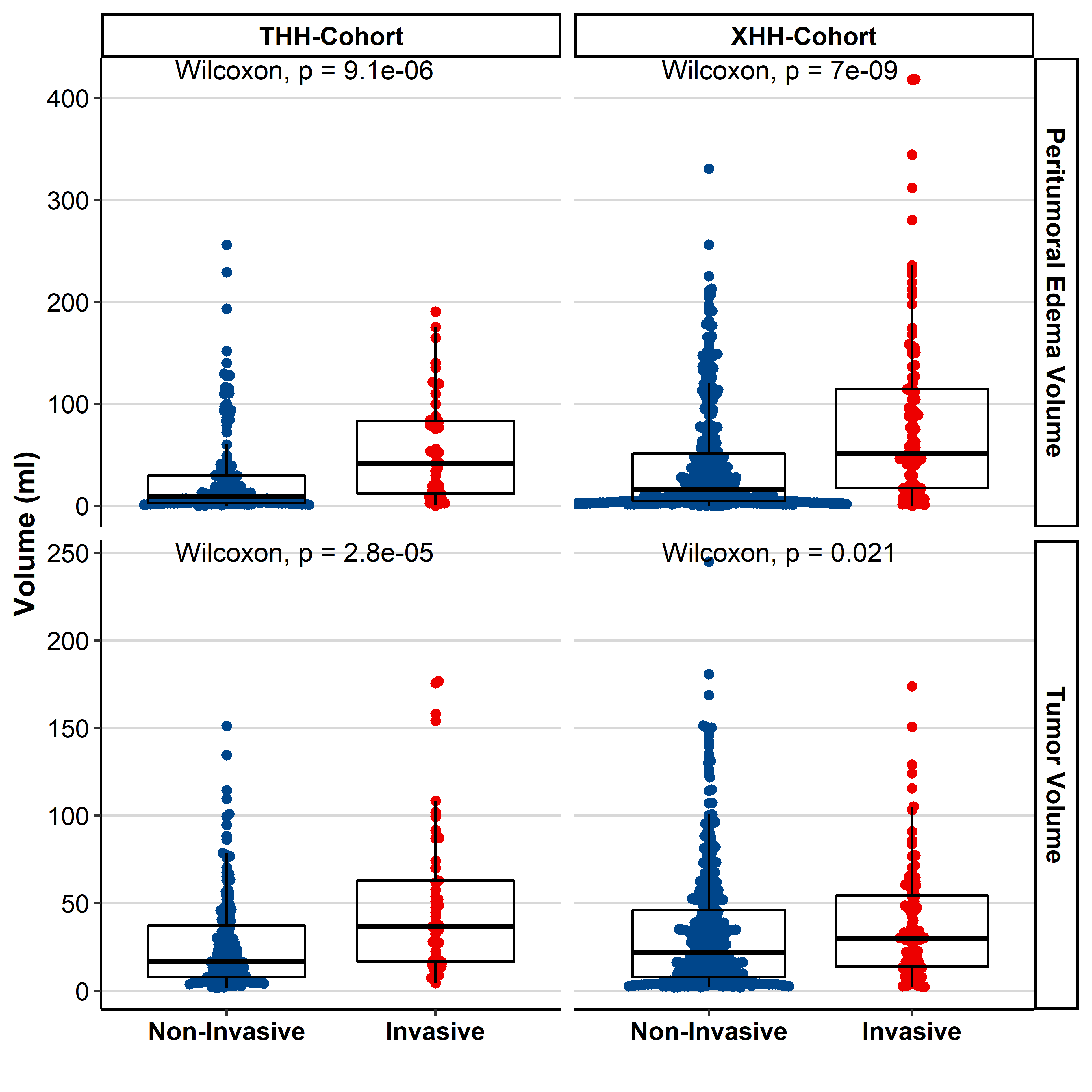

Supplement: Supplementary Figure 1 — Violin plot of the PEV and TV value distribution of different ROI areas in the XHH cohort and THH cohort. PEV, peritumoral edema volume; TV, tumor volume. [file Image_1.tiff]

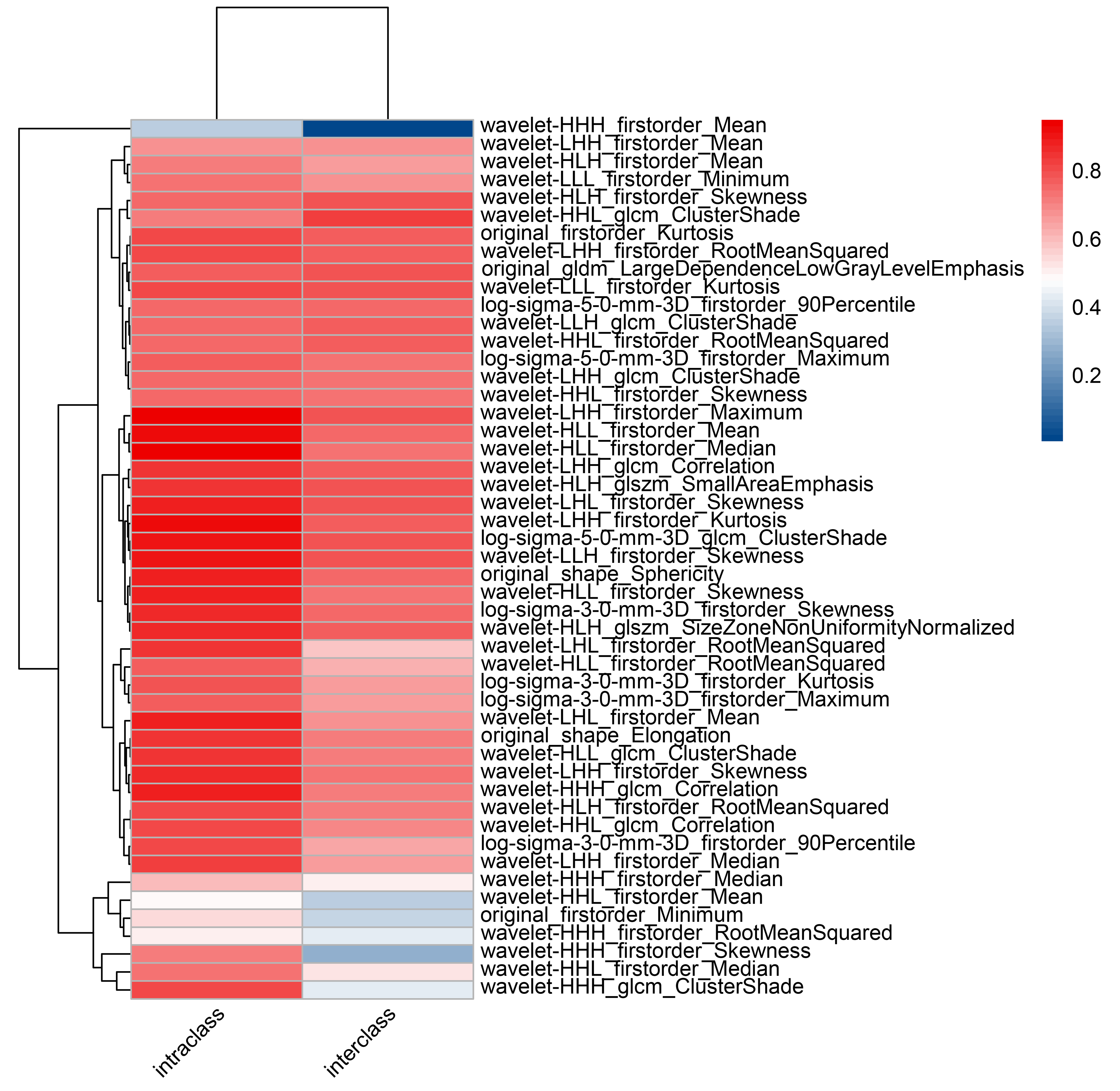

Supplement: Supplementary Figure 2 — Heatmap of radiomics features with an intraclass correlation coefficient <0.8. [file Image_2.tif]

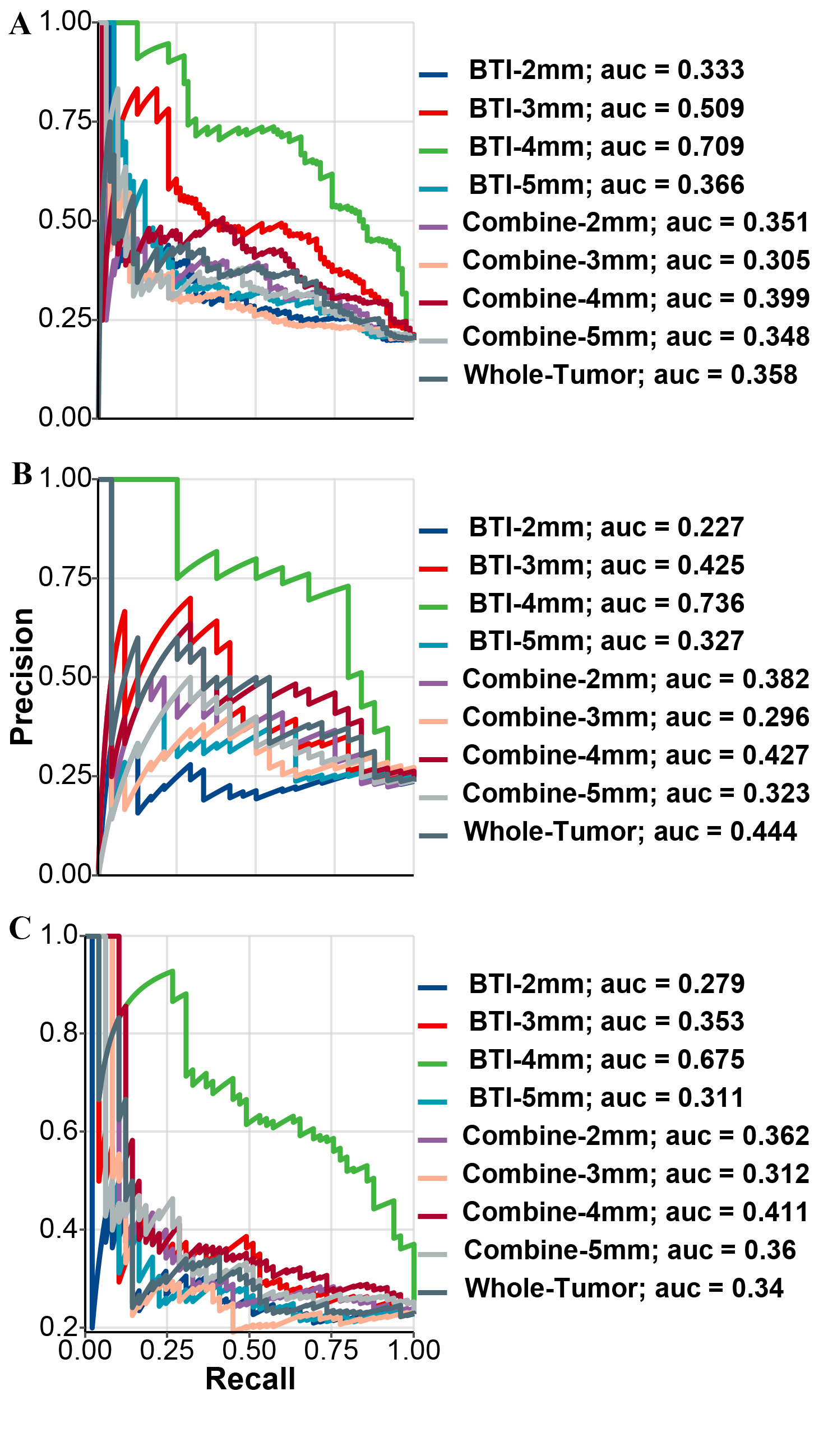

Supplement: Supplementary Figure 3 — The predictive performance of models under the precision-recall curve. Internal training set (A); internal validation set (B); external validation set (C). ****p < 0.0001. [file Image_3.tif]

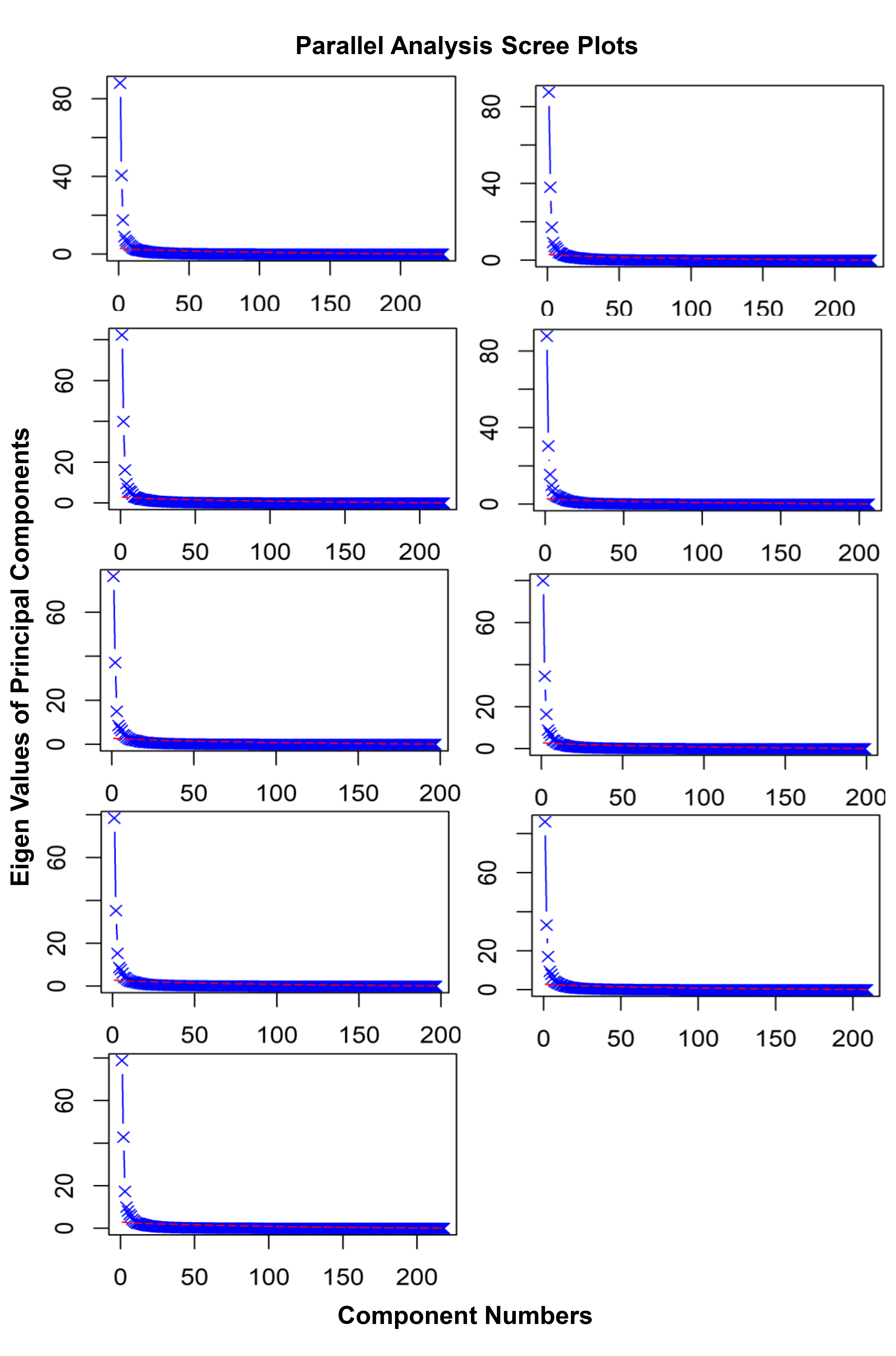

Supplement: Supplementary Figure 4 — Scree plot with parallel analysis for identifying the number of principal components to be kept. The blue cross indicates a principal component, and those above the red dashed line are candidate components. [file Image_4.tif]

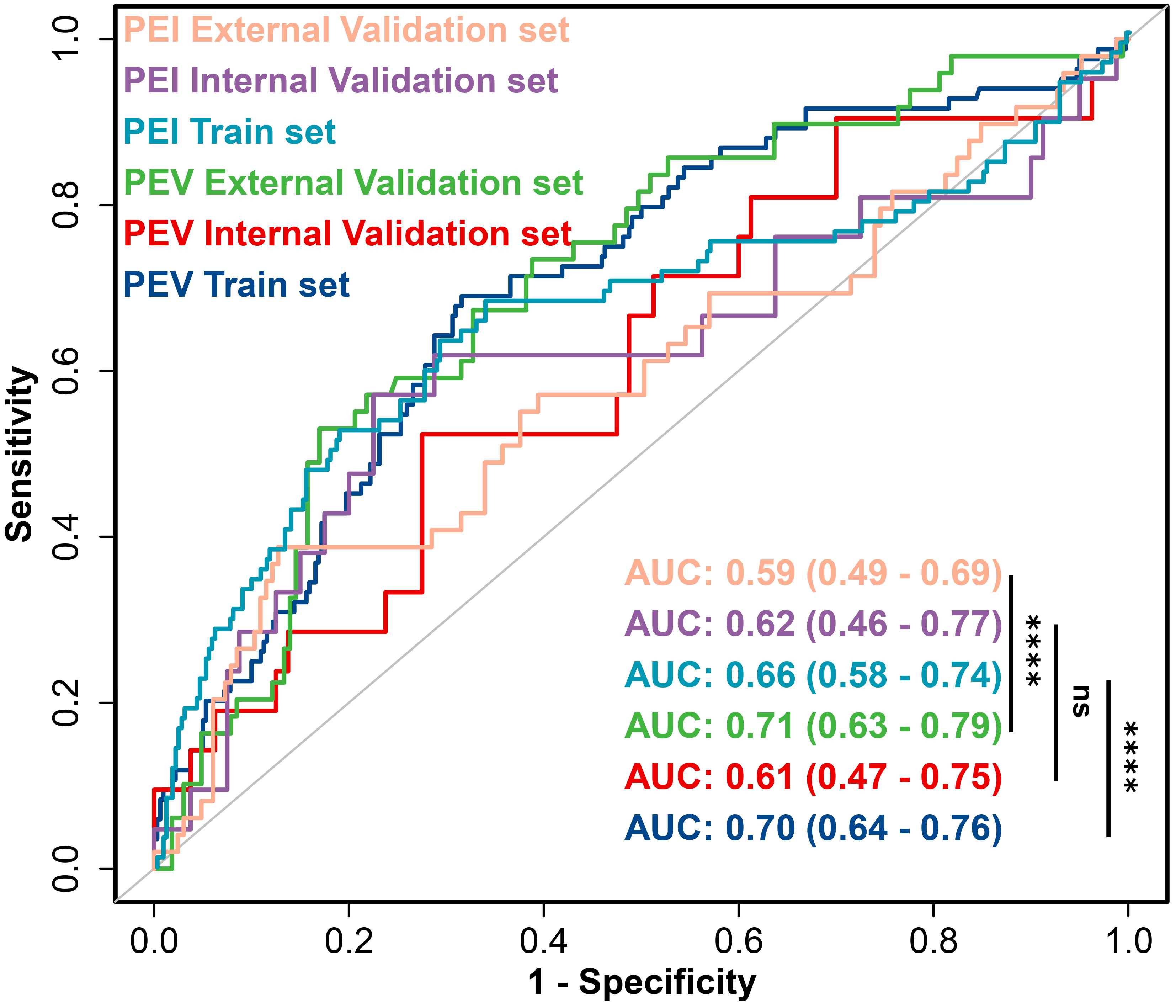

Supplement: Supplementary Figure 5 — The receiver operating characteristic curves of PEV and PEI derived from training and validation sets. ****p < 0.0001. [file Image_5.tif]
